# Improving Secondary Bone Protection Prescription in Patients Admitted With a Femoral Neck Fracture

**DOI:** 10.7759/cureus.18883

**Published:** 2021-10-19

**Authors:** Mohammed Hamid, Anmol Chikhlia, Ashley Gogna

**Affiliations:** 1 General Surgery, University Hospitals Birmingham NHS Trust, Birmingham, GBR; 2 Liver Transplantation and Hepatology, Royal Free Hospital, London, GBR; 3 Trauma and Orthopaedics, Lismore Base Hospital, Lismore, AUS

**Keywords:** geriatrics, orthopaedics, neck of femur fracture, bone-protection, quality improvement tool

## Abstract

Background

The socioeconomic burden caused by fragility fractures is well recognised in today’s ageing society, with hip fractures making a notable contribution. There is a significant national drive for secondary-prevention bone-protection prescription given the high morbidity and mortality rates of femoral neck fractures. A Specific, Measurable, Achievable, Relevant, Time-bound (SMART) aim was constructed to reach the gold standard in a level 2 trauma centre, utilising the Model for Improvement methodology.

Methodology

Baseline data were collected for 79 consecutive patients admitted with a neck of femur fracture. A total of 14% were managed with bone-protection plans. The root cause analysis identified three elements having a major impact on the prescription of secondary bone-protection medication: the lack of awareness, education, and a structured multidisciplinary team (MDT) approach. Appropriate plan-do-study-act cycles were implemented and change audited.

Results

Following cycles one and two, the mean percentage of patients managed with bone-protection plans increased from 14% to 44% and 76%, respectively. A statistical process control chart demonstrated positive change for each cycle, with p-values of <0.01 and <0.001, respectively. After our final cycle, 100% of patients suffering from a femoral neck fracture were being managed with appropriate bone-protection plans according to the Royal College of Physicians’ national hip fracture database. We observed 100% sustainability two years later, despite the coronavirus disease 2019 pandemic service disruptions and redeployment of staff.

Conclusions

Departmental awareness and education played an important role in this quality improvement project. The ultimatum and sustainability intervention was ‘responsibility charting’ among the MDT: setting clear roles within the team to deliver better patient care.

## Introduction

Approximately 70,000 hip fractures occur annually in the United Kingdom, with care totalling over two billion pounds [1]. Hip fractures are a serious consequence of falls in the elderly, with a mortality of 10% at one month and 30% at one year [2,3]. Sustaining a fragility fracture at least doubles the risk of future fractures [1], with up to 11% of patients sustaining a second fracture in the first year [4,5].

Therefore, secondary prevention of hip fractures is of great importance. Unless contraindicated, all patients sustaining a hip fracture should be treated with bisphosphonates [6], which reduces the relative risk of recurrent fractures by up to 35% [7]. When bisphosphonates are contraindicated, other medical treatments in secondary prevention can be used, including denosumab, vitamin D, and calcium supplementation.

Problem

In this study, baseline data were collected over a two-month period between September and November 2018. A total of 79 patients were admitted with a neck of femur fracture (NOF) to our orthopaedic wards. Data analysis found that 13.9% (n = 11) of our patients were assessed for the need for secondary bone-protection prescription. Of these patients, nine received a form of bone-protection medication, and the remaining two patients were deemed clinically unsuitable for treatment. Out of the nine patients receiving medication, 1.3% (n = 1) were prescribed a bisphosphonate, with 10.1% (n = 8) prescribed other bone-protection measures. The majority, 86.1% (n = 68), were neither assessed for nor prescribed bone protection, including 3.8% (n = 3) of patients who were already on secondary-prevention bone-protection medication pre-admission. Thus, the lack of an effective secondary-prevention bone-protection management plan for all patients suffering from a hip fracture was noted in a large district general hospital, housing a level two trauma centre.

Deficiency in this practice has been documented yearly across the United Kingdom by the National Institute of Clinical Excellence (NICE) [8], the National Fracture Liaison Service Database (FLS-DB) [9], and the National Hip Fracture Database (NHFD) [10]. The local baseline of 13.9% in 2018 fell below the national average of 36% recorded by the FSL-DB, which evaluated 58,979 patients in 2018 [9].

Aim

A Specific, Measurable, Achievable, Relevant, Time-bound (SMART) aim was devised to improve the rate of secondary-prevention bone-protection assessment and prescription in patients admitted with a NOF to our orthopaedic wards, by an additional 40% per year towards the 100% gold standard, utilising the Model for Improvement methodology [11].

## Materials and methods

This quality improvement (QI) project did not require local ethical approval. Our documentation is in line with the Standards for QUality Improvement Reporting Excellence (SQUIRE) guidelines [12].

The initial audit captured data from the patients’ clerking sheets, their clinical notes, and their prescription charts during the first week of admission. The first dataset collected other equally essential information for comparison (n = 79): Whether initial blood tests were taken? (96.2%); did the patient have a chest X-ray? (96.2%); did the patient have an electrocardiogram (ECG)? (89.9%); whether the patients had their venous thromboembolism (VTE) assessment done on admission? (96.2%).

During the root-cause analysis stage, several QI methods were trialled to understand the reasons behind the low number of bone-protection plans. These included the five-whys system, the use of fishbone, and affinity diagrams; however, process mapping and driver diagrams were found to be the most useful to solve this aspect of care [11].

Process mapping

**Figure 1:**
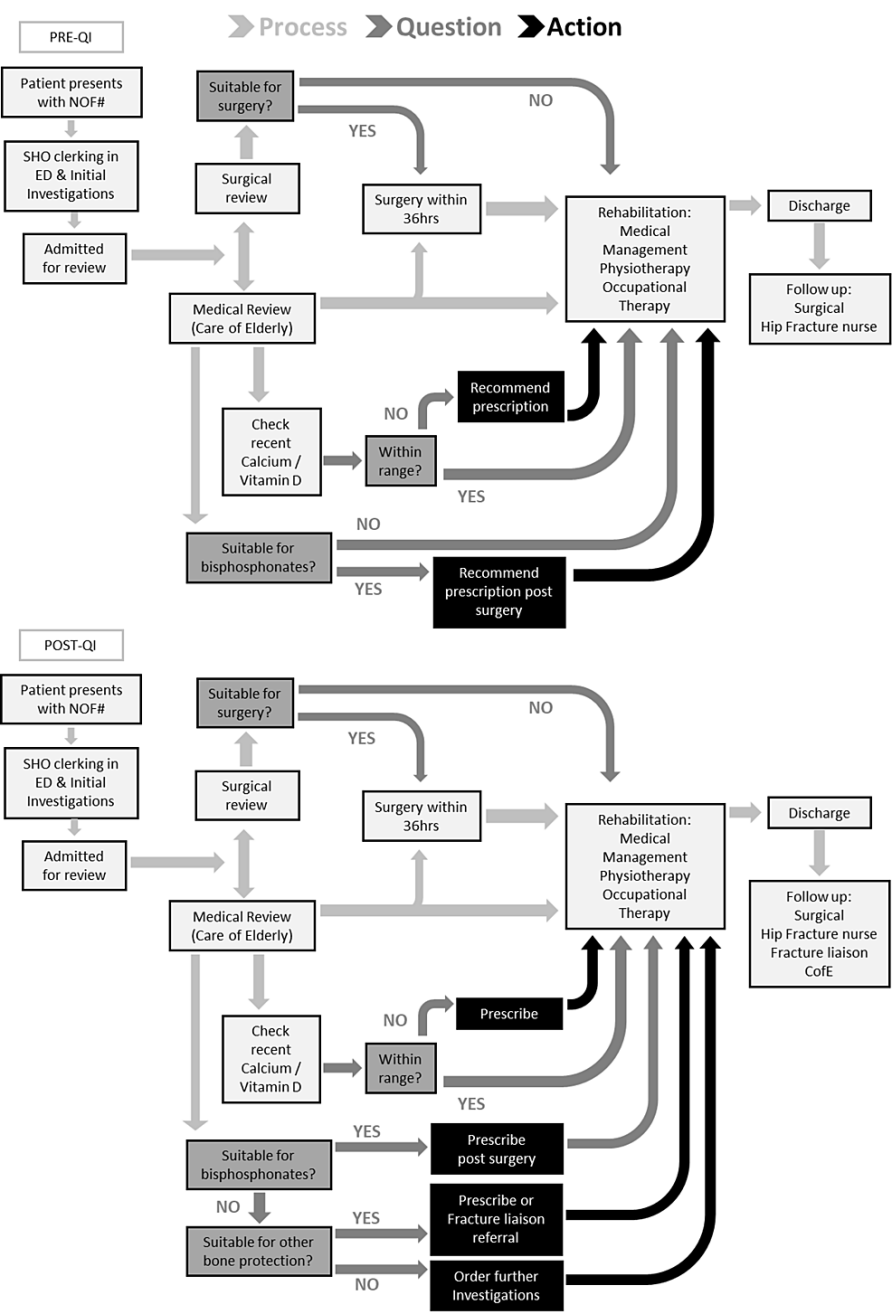
The change in the process map before (top) and after (bottom) the QI project. Responsibility charting led to the consolidation of roles among the MDT to tackle the lack of bone-protection assessment, plan, and prescription. SHO: Senior House Officer; ED: Emergency Department; CofE: Care of Elderly Physicians; QI: quality improvement; MDT: multidisciplinary team

The two-process maps shown in Figure 1 delineate the journey taken by patients sustaining a hip fracture from admission to discharge before (top) and after (bottom) the QI project cycles. These flow diagrams present the stages and the stakeholders involved in secondary-prevention bone-protection assessment and prescription. It is evident from the top process map that the first clinicians to see the patients are the Trauma and Orthopaedic Senior House Officers (SHO) in the Emergency Department (ED) during clerking. Patients are then admitted onto the ward and assessed by a care of the elderly physician (CofE), that is, a geriatrician, before undergoing surgery within 36 hours. Patients who are deemed not fit for surgery at this stage are considered for conservative management. For most of the day, the trauma and orthopaedic wards are generally manned by junior doctors with external support available from the CofE team on bone-protection prescription recommendations. The patients then undergo rehabilitation prior to discharge, with contact from the hip fracture nurses, whose role involved following up the patients’ progress, monitoring their bone-protection needs (such as the necessity for a dual-energy X-ray absorptiometry [DEXA] scan prior to prescription), and updating the NFHD [13].

Local drivers

By scrutinising the different stages and stakeholders involved in the top process map during forums, we were able to attain reasons (drivers) pertaining to the failure of local practice; some or all of which are likely to exist nationally. It was made apparent during meetings that there was a lack of a robust communication portal between all the following stakeholders.

Junior doctors clerking patients in ED do not feel comfortable assessing patients for bone protection due to the lack of knowledge. During forums, they requested a more succinct clerking proforma and more senior support as bone protection was often skipped due to the busy nature of their on-call shifts.

Ward doctors also expressed no awareness or the knowledge required for bone-protection assessment and prescription. They also exclaimed that the responsibility of this task had never been made apparent and they always relied heavily on contacting the CofE, hip fracture nurses, and fracture liaison for prescription advice, adding more to their workload.

The CofE team who provided the majority of the recorded baseline assessments routinely provided recommendations for the ward team without fulfilling the plan themselves. The main reason for this was the frequent movement of patients between different departments and the delay in blood results (e.g., vitamin D).

Hip fracture nurses and fracture liaisons would often require a confirmation from the CofE plan before giving further advice, but were unable to prescribe medication.

Primary drivers

**Figure 2:**
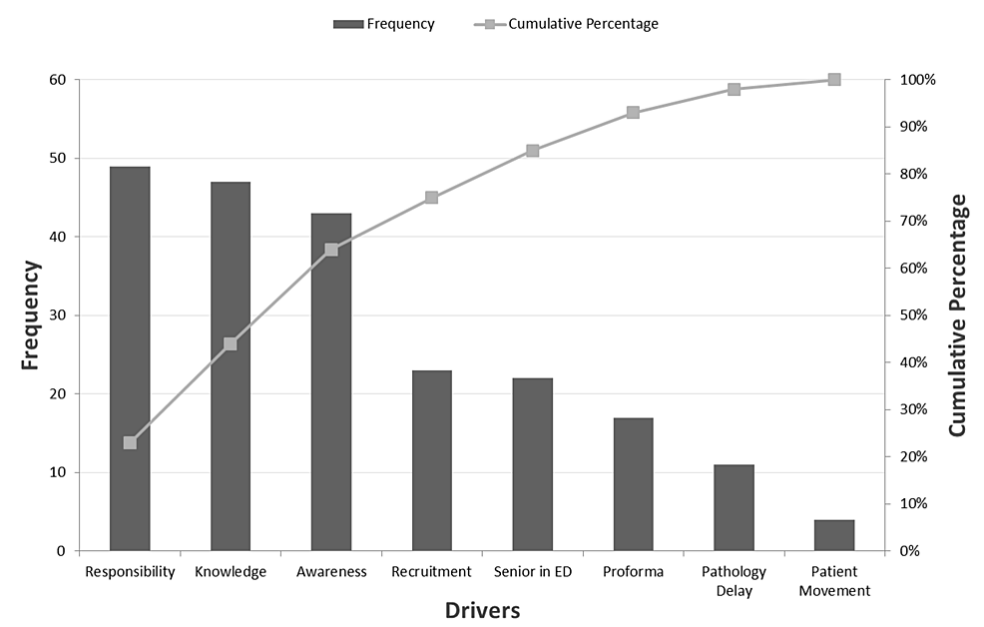
Pareto chart demonstrating the frequency and cumulative percentage of the drivers impacting bone-protection plans for the baseline data, as gathered from a stakeholder survey. Awareness, education (knowledge), and responsibility represent the main drivers. ED: Emergency Department

The local drivers documented above were tallied by frequency of occurrence through a stakeholder survey. The Pareto rule [14] was then applied to focus the team’s effort on the three primary drivers with the most influence (Figure 2).

First, the lack of awareness surrounding secondary-prevention bone-protection among junior ward doctors, the department, patients, and their relatives. Second, the lack of specific education provided to ward doctors on bone-protection assessment and prescription during their job induction. Finally, the lack of an organised structure among the multidisciplinary team (MDT) surrounding this element of care impacting responsibility.

It was recognised that the lack of awareness among the regular duty doctors looking after this population of patients stemmed from the lack of education and unstated responsibility of bone-protection prescription on their job roles. Junior doctors rotate every four to six months between different specialities and rely on their induction to provide guidance on their duties. However, no information was provided on this aspect of care. Forum feedback also highlighted that this element of care seemed ‘difficult’ for the junior doctors as patient suitability often differed and the CofE team’s clinical judgment was required most of the time. Awareness among the department regarding the outcomes of secondary-prevention bone-protection management was also scarce despite the availability of the Royal College of Physicians’ NHFD. Another factor highlighted within the structure of this MDT was that there were two different hip fracture nursing groups: the ward hip fracture nurses were part of the orthopaedic team, and the fracture liaison nurses supported the entire hospital. The roles and responsibilities of these two teams often overlapped introducing ‘waste’ into the system.

Patient involvement

During the initial data collection stage, 89.9% (n = 71) of the patients sustaining a NOF, and their relatives, were unaware of the availability and benefits of secondary bone-protection management, as indicated by a face-to-face survey. This highlighted that patient and public involvement was required. After considering this aspect during our strategy meeting, an awareness poster was created.

Strategy

Once the above key drivers were identified, a few appropriate plan-do-study-act (PDSA) cycles were implemented to achieve the study’s aim. Each cycle was implemented in succession, with a fortnight of data collection commencing each action.

PDSA 1, Poster, January 2019

To tackle the lack of awareness among the junior ward doctors, the department, and the patients, an informative poster was disseminated within the trauma and orthopaedic departments to reintroduce awareness surrounding the issue of bone-protection assessment and prescription (Appendix). This poster was displayed in the handover room and in front of the hip fracture nurses’ desks, in the ED where patients get clerked, and on the wards where patients were being looked after by the regular team. The posters were also made available for patients and their families to see.

PDSA 2, Education Session, March 2019

To inform the regular duty doctors on when and why it is appropriate to prescribe bone protection, an educational session was conducted. The session targeted both the acute take doctors providing the initial clerking and the ward doctors maintaining their care. The material was transferred onto an induction document and provided to new doctors joining the department.

PDSA 3, MDT Meeting, April 2019

Using responsibility charting, local job descriptions were adjusted to meet the needs of patients sustaining a hip fracture. At this meeting, the roles and responsibilities of both assessing and prescribing secondary-prevention bone-protection were handed over to the CofE team, with management support from the hip fracture nurses and specialist prescriptions managed by the fracture liaison team (Figure 1, Post QI).

## Results

**Figure 3:**
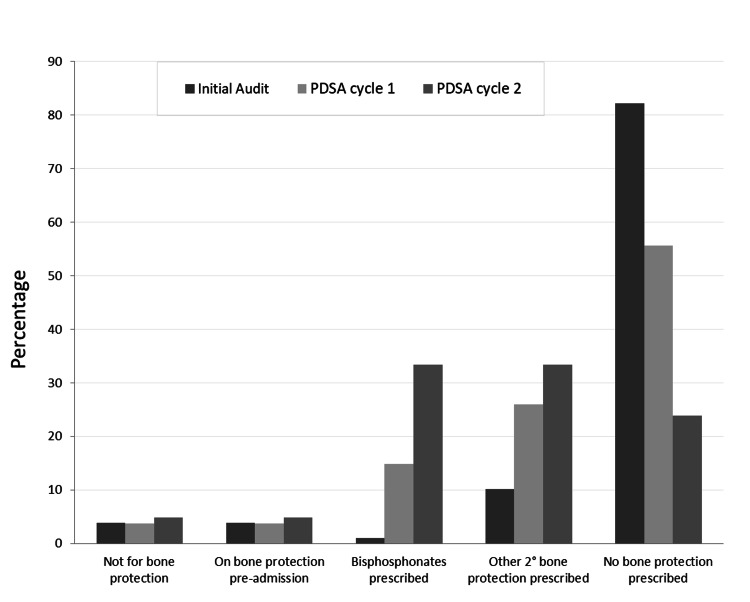
The improvement in bone-protection assessment and prescription as a percentage, comparing baseline data up to PDSA cycle 2. PDSA: plan-do-study-act

The mean age of all patients across this study was 81 years. Female patients made up 54% of the studied population. From a baseline of 14%, the proportion of patients being assessed for and/or prescribed bone-protection medication increased to 44% and subsequently to 76% after the first and second PDSA cycles, respectively. Figure 3 demonstrates the change in prescribing practice over the course of the first two PDSA cycles.

During the first PDSA cycle, data from 27 consecutive patients admitted with a NOF were collected. Of these, there was a significant increase in patients assessed and/or prescribed bone protection to 44.4% (n = 12, p < 0.01). An increase in the proportion of patients being prescribed bisphosphonates or other secondary bone protection was observed to be 14.8% (n = 4) and 25.9% (n = 7), respectively. One patient was assessed and deemed not suitable for bone-protection medication. Out of the 55.6% (n = 15) who did not have any bone-protection prescribed, one patient was on bone protection prior to admission.

During the second PDSA cycle, data from 21 consecutive patients sustaining a NOF were collected. There was a further significant improvement in the proportion of patients being assessed and/or prescribed bone protection to 76% (n = 16, p < 0.001). The proportion for bisphosphonates and other secondary bone-protection prescription both increased to 33.3% (n = 7). Again, one patient was assessed and deemed not suitable for bone-protection medication. One patient was on bone protection prior to admission; however, on this occasion, the patient was assessed and had a bone-protection plan put in place. There was a drop to 23.8% (n = 5) of patients who were not assessed or had any bone protection prescribed.

Following the April 2019 MDT meeting, data from the Royal College of Physicians NHFD for the hospital were reviewed. On average, one out of ten patients were being assessed or placed on a bone-protection plan prior to the QI, not far from our recorded 14%. There was a significant improvement to all patients being assessed or placed on a bone-protection plan a fortnight after the MDT meeting, PDSA cycle 3 (n = 50, 100%).

**Figure 4:**
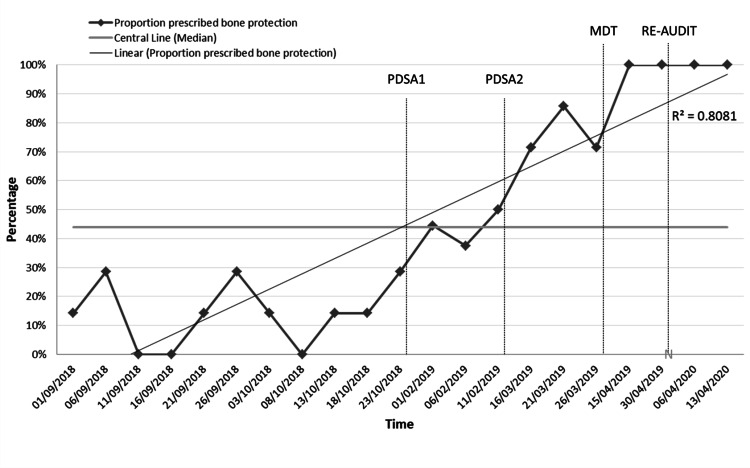
SPC run chart of the project, with a median control line and a coefficient of determination (R²) trend line. The timing of the first two PDSA cycles, the subsequent MDT meeting (last PDSA cycle), and the annual re-audit are signposted. Overall, regression modelling displays a positive significant shift (>0.7), as well as many points below and above the median line, indicating non-random variation and corroborating statistical significance. SPC: statistical process control; PDSA: plan-do-study-act; MDT: multidisciplinary team

An annual re-audit of 50 patients amid the peak of the coronavirus disease 2019 pandemic, which led to major service disruptions and redeployment of staff, showed no waiver in the established practice, demonstrating sustainability. Figure 4 represents the progress made during this QI project; data were not continuously collected between PDSA cycles making it difficult to evaluate how soon after a PDSA cycle the improvement in the rate for bone-protection prescription occurred.

## Discussion

This QI project demonstrates the significance of penetrating change through all layers of the MDT. While significant improvements were exhibited after the first two PDSA cycles, which targeted awareness and education, the ultimatum was realised with direct engagement of the MDT. Clarifying the roles of the CofE team, the hip fracture, and fracture liaison nurses enabled ‘responsibility charting’ [15]. This has been described by the NHS Improvement as a tool to clarify any confusion, assumptions, and misunderstandings; prevent duplication of effort; and disclose issues such as gaps in responsibility and miscommunication [15]. The NHS devised a model in which the roles of ‘the responsible’ (individual completing the task), ‘the accountable’ (individual answerable to the task), ‘the consult’ (individuals providing advice), and ‘the informed’ (individuals who require to take action as a result of the outcome) are identified [16]. For the prescription of bone protection, this QI project identified ‘the responsible’ as the care of elderly physicians who now prescribe bone protection or refer to the fracture liaison team; ‘the accountable’ as the consultant under whom the patient is admitted; ‘the consult’ as the ward doctors and pharmacists who can now provide advice as per their induction training and CofE plan; and ‘the informed’ as the hip fracture nurses and patient’s general practitioner (GP) who follow-up on the patient’s secondary bone-protection management. Consequently, the rate of bone-protection prescription increased significantly.

The success of this project is reflected in the Royal College of Physicians NHFD with our department reaching the gold standard [13]. The anticipated clinical and financial implications of this are vast, with a theoretical reduction in secondary fractures and the subsequent associated costs [1,7]. Secondary prevention can be argued to be of greater value than primary prevention, with 50% of all hip fractures occurring in those previously sustaining a fracture [17-20]. In addition, it has been noted that the same reduction in fracture incidence through primary prevention would necessitate the identification of five to six times more patients [21,22].

To ensure sustainability, the roles and responsibilities, as per the ‘responsibility charting’ tool, are clarified at departmental inductions to inform rotating doctors. The educational session and departmental poster are also provided at inductions to serve as reminders.

Limitations

A limitation of this project was that patients were not stratified by age. NICE guidelines state that patients sustaining fragility fractures over 60 years of age and who are not frail should be evaluated for osteoporosis by measurement of bone mineral density before commencing bisphosphonates, while patients over 75 years of age sustaining fragility fractures can be commenced on bisphosphonates without the need for DEXA scanning [23]. There is minimal guidance on whether this should be the case for other bone protection treatments. Despite this, several studies have suggested that a fragility fracture in itself is a sign of osteoporosis and should automatically warrant bone-protection prescription [24,25]. In this study, while we assessed whether a patient was not eligible for bone protection, we did not examine whether this was due to the patient’s age, or whether this was due to another contraindication to treatment instead.

We recognised that data were mainly collected during the first week of the patients’ admission. Therefore, bone protection may have been prescribed later during the admission and not captured in our analysis of the bone-protection prescriptions. Nevertheless, the care of elderly physician’s assessment would almost always take place in the first week of admission, and a recent departmental audit showed the length of stay for a traumatic hip operation averaging seven to eight days; thus, suggesting that collecting data during the first week of admission was most likely sufficient.

Future work

We will continue to re-audit annually to ensure sustainability is maintained at 100% and seek to investigate if a fall in standards is found.

In addition to the prescription of bone-protection agents, persistence with therapy is another factor that is imperative to address. Studies have shown that among patients on daily and weekly bisphosphonate treatment, only approximately 30% and 40%, respectively, persist with treatment at one year [26]. A study in France evaluating secondary prevention of osteoporotic fractures found that one of the main causes of discontinuation of treatment was the non-renewal of prescriptions by the patient’s GP [27]. The Royal College of Physicians recommends that acute hip fracture teams follow up their patients over 120 days. Over this course, they should assess persistence with bone protection. The 2018 National Hip Fracture Database Report found that less than 40% of patients were being followed up over this duration to confirm they were still taking bone-protection medication [9]. Future work to follow up patients on bone protection may reveal whether ‘responsibility charting’ can be expanded to primary care services to ensure continuity of bone-protection treatment.

Implications

The last FSL-DB update in 2018 showed a national average of 36% compliance for this practice [9]. Trauma centres across the nation can adopt our approach to improve services, patient care, and contribute to cost savings. We anticipate that the step-by-step methodology utilised for this project can also be successfully replicated for other healthcare issues needing to be addressed.

## Conclusions

This project demonstrates how using the Model for Improvement methodology can impact the rate of secondary bone-protection assessment and prescription in patients admitted with a NOF. PDSA cycles to increase awareness and education played a pivotal role in improving local practice. However, the ultimatum and sustainability factor was ‘responsibility charting’ among the MDT: setting clear roles within the team to deliver gold-standard patient care.

## Figures and Tables

**Figure 5:**
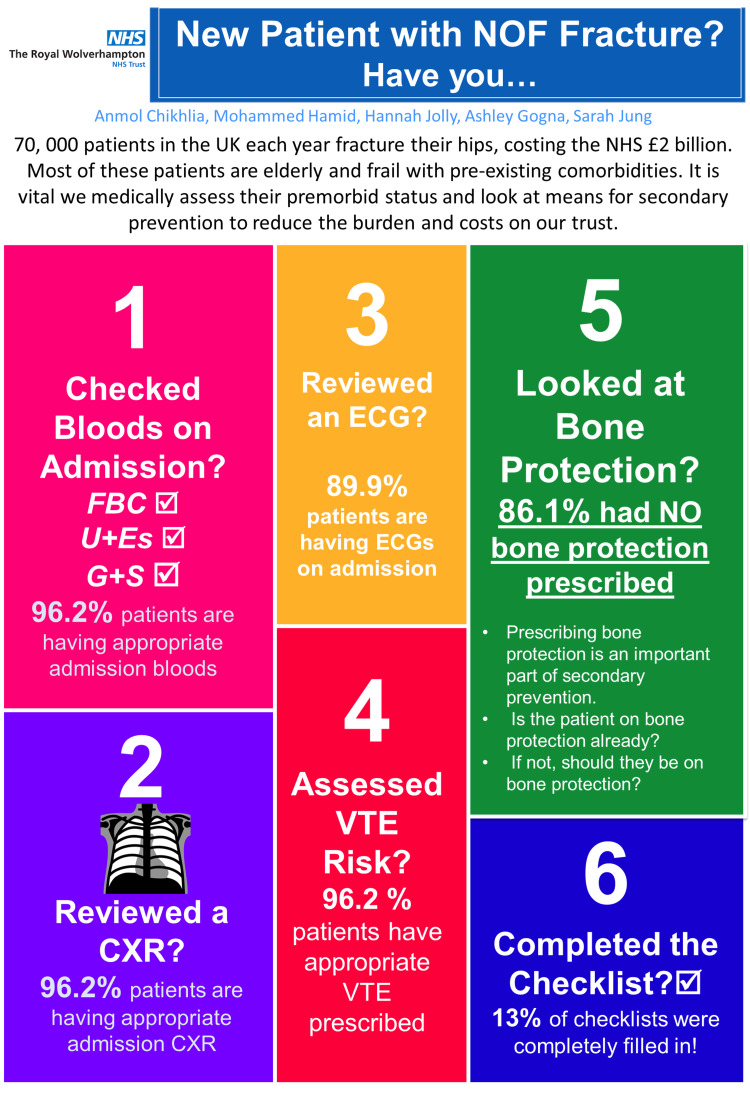
For the first PDSA cycle intervention, this poster was displayed across the trauma and orthopaedic departments to increase awareness of the current practice of secondary-prevention bone-protection prescription among patients admitted with a NOF. PDSA: plan-do-study-act; NOF: neck of femur fracture
